# Association between increased serum interleukin-8 levels and improved cognition in major depressive patients with SSRIs

**DOI:** 10.1186/s12888-023-04616-z

**Published:** 2023-02-23

**Authors:** Yuan Cai, Zhen Hua Zhu, Rong Hua Li, Xu Yuan Yin, Ru Feng Chen, Li Juan Man, Wen Long Hou, Hong Liang Zhu, Jing Wang, Huiping Zhang, Qiu Fang Jia, Li Hui

**Affiliations:** 1 School of Mental Health, Wenzhou Medical University, Wenzhou, 325035 Zhejiang People’s Republic of China; 2 Research Center of Biological Psychiatry, Suzhou Guangji Hospital, Medical College of Soochow University, No. 11 Guangqian Road, Suzhou, 215137 Jiangsu People’s Republic of China; 3 Departments of Psychiatry and Medicine, Boston University Chobanian and Avedisian School of Medicine, Boston, MA 02118-2526 USA

**Keywords:** Major depressive disorder, Interleukin-8, Cognitive function, SSRIs

## Abstract

**Background:**

The effect of neuroinflammatory cytokines on cognitive deficits in patients with major depressive disorder (MDD) can be altered by selective serotonin reuptake inhibitors (SSRIs). This study aimed to examine serum interleukin-8 (IL-8) levels, cognitive function, and their associations in MDD patients with SSRIs.

**Methods:**

Thirty SSRI-treated MDD patients and 101 healthy controls were recruited for this study. We examined cognitive performance using the Repeatable Battery for the Assessment of Neuropsychological Status (RBANS) and serum IL-8 levels using the Human Inflammatory Cytokine Cytometric Bead Array in both cases and controls.

**Results:**

The RBANS test scores were significantly lower in MDD patients with SSRIs than in healthy controls after controlling for covariates (all *p* < 0.001). Serum levels of IL-8 were higher in MDD patients with SSRIs than in healthy controls after adjusting for covariates (*F* = 3.82, *p* = 0.05). Serum IL-8 levels were positively correlated with sub-scores of delayed memory (*r* = 0.37, *p* = 0.04) and visuospatial/constructional (*r* = 0.43, *p* = 0.02) in MDD patients with SSRIs but not in in healthy controls (delayed memory score: *r* = -0.12, *p* = 0.24; visuospatial/constructional score: *r* = 0.02, *p* = 0.81).

**Conclusions:**

Our findings suggested that increased serum IL-8 level might not only be involved in the MDD psychopathology or the use of SSRIs but also correspond to improving MDD delayed memory and visuospatial/constructional function.

**Supplementary Information:**

The online version contains supplementary material available at 10.1186/s12888-023-04616-z.

## Background

Cognitive deficits have been regarded as the core features of major depressive disorder (MDD) [1–3]. Previous studies indicated that cognitive deficits of MDD mainly occurred in the following cognitive domains, such as memory, attention, visuospatial/constructional skills, language, executive function and processing speed [2, 4–6]. Moreover, cognitive deficits may further influence treatment, rehabilitation, quality life, social activity, and even employment for MDD [7]. Thus, cognitive deficits should be considered a potential target for the treatment and rehabilitation of MDD [8–10]. However, the pathogenesis of cognitive deficits in patients with MDD is still not well understood and requires further investigation.

Interleukin 8 (IL-8) is an inflammatory cytokine synthesized and released by macrophages and brain microglia and astrocytes [11, 12]. IL-8 may serve either pro- or anti-inflammatory role mainly depending on the concentration [13]. An animal study has shown a higher expression level of *IL-8* gene in microglia and astrocytes of the hippocampus compared to other brain regions [14]. Selective serotonin reuptake inhibitors (SSRIs) have been reported to modulate the ability of microglia and astrocytes to produce inflammatory cytokines [15–20]. Altered activity of hippocampal microglia and astrocytes may contribute to cognitive deficits and be associated with the decline in cognitive performance in patients with MDD [21]. Mounting evidence support that MDD is considered to be a neuroinflammatory disorder that is closely related to abnormal activation of microglia and astrocytes [12, 22, 23]. Previous studies demonstrated the immunomodulating effects of SSRI treatment on MDD, further suggesting that SSRIs may possess anti-inflammatory properties [24, 25]. Moreover, several studies have found that serum IL-8 influenced cognitive function in normal elderly subjects and patients with mild cognitive impairment (MCI) or Alzheimer’s disease (AD) [5, 26–30]. Thus, the above findings suggest that serum IL-8 may be implicated in regulating cognitive performance in MDD patients with the administration of SSRIs.

However, no studies have examined serum IL-8 levels in relation to cognitive function in MDD patients with SSRIs. Thus, the present cross-sectional study aimed to examine serum IL-8 levels, cognitive function, and their correlations in MDD patients with SSRIs.

## Methods

### Subjects

The present study was performed during the time period from August 2017 to April 2021. Following a complete description of the study protocol and procedure to each subject by a research coordinator or a clinical psychiatrist, the signed informed consent was obtained in accordance with the study protocol that was approved by the Institutional Review Board of Suzhou Guangji Hospital, Medical College of Soochow University. MDD Patients with SSRIs (*n* = 30; male/female = 11/19) and Healthy controls (*n* = 101; male/female = 52/49) came from the part of subjects of recent study of our research group [31].

### Cognition an IL-8 assessment

Cognitive performance was assessed using the Repeatable Battery for the Assessment of Neuropsychological Status (RBANS) [32]. The RBANS assessment process was described in our previous studies [33, 34]. The English RBANS version has been translated into Chinese, and its clinical effectiveness and reliability were established in patients with schizophrenia and healthy controls [35]. The handing process of blood sample and serum IL-8 assessment method also have been descried in our recent study [31].

### Statistical analysis

The demographic and clinical variables were compared between MDD patients with SSRIs and healthy controls using an analysis of variance (ANOVA) for continuous variables and a Chi-squared test for categorical variables. Serum IL-8 levels were not normally distributed and were thus log-transformed. We compared RBANS test scores and serum log_10_IL-8 levels between case and control groups using an ANOVA. When significant differences were observed, sex, age, education, smoking, drinking, marriage and BMI were added as covariates. The relationships between serum log_10_IL-8 levels and RBANS test scores in MDD patients with SSRIs as well as in healthy controls were evaluated with Pearson's product moment correction coefficients, respectively. Continuous data were presented as the mean and standard deviation (mean ± SD), and all *p* values were 2-tailed at a significance level of < *0.05*.

## Results

### Demographic and clinical characteristics

There were no significant differences in sex, age, smoking, drinking, marriage, and BMI between MDD patients with SSRIs and healthy controls (Table 1). However, education was significantly different between cases and controls (*F* = 21.87, *P* < 0.001). The mean and SD of illness duration, age of onset, hospitalization number, and HAMD score in MDD patients with SSRIs were 3.90 ± 4.27 years, 36.59 ± 14.80 years, 1.52 ± 0.87, and 22.03 ± 6.49, respectively.

**Table 1:** Demographic and clinical data of MDD patients with SSRIs and healthy controls

Variables	MDD Patients with SSRIs	Healthy Controls	F or χ^2^	p
	*N* = 30, Mean (SD)	*N* = 101, Mean (SD)		
Gender (male/female)	11/19	52/49	1.48	0.22
Age (years)	39.73 (15.66)	37.38 (12.13)	0.76	0.39
Education (years)	10.07 (3.39)	13.00 (2.90)	21.87	**< 0.001**
Marriage (unmarried/married/divorced)	8/21/1	31/68/2	0.11	0.95
BMI (kg/m^2^)	22.51(3.07)	22.94 (3.03)	0.43	0.51
Smoking (smoker/nonsmoker)	27/3	76/25	1.96	0.16
Drinking (drinker/nondrinker)	26/4	92/9	0.13	0.72
Age of Onset (years)	36.59 (14.80)	-	-	-
Hospitalization Number	1.52 (0.87)	-	-	-
Duration of Illness (years)	3.90 (4.27)	-	-	-
HAMD Score	22.03 (6.49)	-	-	-

*Abbreviations: MDD* Major depressive disorder, *SD* Standard deviation, *SSRIs* Selective serotonin reuptake inhibitors, *BMI* Body mass index, *HAMD* Hamilton Depression Scale

### Comparisons of RBANS scores and log_10_IL-8 levels between two groups

**Fig. 1:**
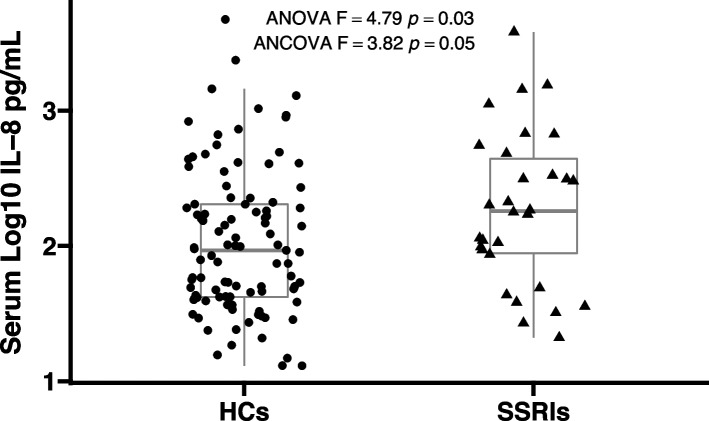
Comparison of serum log_10_IL-8 levels between MDD patients with SSRIs and healthy controls. Serum log_10_IL-8 levels in MDD patients with SSRIs were nominally higher in comparison to healthy controls after adjusting for covariates (2.27 ± 0.57 vs 2.03 ± 0.53, *F* = 3.82, *p* = 0.05). Abbreviations: IL-8, interleukin-8; ANOVA, analysis of variance; ANCOVA, analysis of covariance; HCs, healthy controls

The mean and SD of all RBANS test scores in 30 MDD patients with SSRIs and 101 healthy controls were shown in Table 2. There were significant differences in all RBANS test scores between these two groups (all, *p* < 0.001). The difference remained significant after controlling for sex, age, education, marriage, smoking, drinking, and BMI (all, *p* < 0.001). As shown in Fig. 1, serum log_10_IL-8 levels were significantly higher in MDD patients with SSRIs than healthy controls (2.27 ± 0.57 vs. 2.03 ± 0.53, *F* = 4.79, *p* = 0.03). When sex, age, education, marriage, smoking, drinking, and BMI were included in ANOVA as covariates, a nominally significant difference between cases and controls was observed (*F* = 3.82, *p* = 0.05).

**Table 2:** Comparisons of the RBANS test scores between MDD patients with SSRIs and healthy controls

Index	MDD Patients with SSRIs (*n* = 30), Mean (SD)	Healthy Controls (*n* = 101), Mean (SD)	F	*p*	Adjusted *F* value ^a^	Adjusted *p* value ^a^
Immediate Memory	63.03(16.70)	90.74(15.35)	72.36	**< 0.001**	79.66	**< 0.001**
Visuospatial/Constructional	65.67(9.40)	82.03(12.01)	47.03	**< 0.001**	52.24	**< 0.001**
Language	70.03(14.50)	96.30(13.49)	84.66	**< 0.001**	98.25	**< 0.001**
Attention	90.00(19.60)	110.71(13.73)	42.69	**< 0.001**	59.68	**< 0.001**
Delayed Memory	69.40(16.71)	93.30(7.38)	125.82	**< 0.001**	133.71	**< 0.001**
RBANS Total Score	65.20(12.33)	92.17(9.56)	160.14	**< 0.001**	239.06	**< 0.001**

*Abbreviations: RBANS* the repetitive battery for the assessment of neuropsychological status, ^a^ Adjusted* p* value indicated the F and *p* value after adjusting for age, gender, education, marriage, BMI, smoking, and drinking

### Associations between RBANS scores and log_10_IL-8 levels

**Fig. 2:**
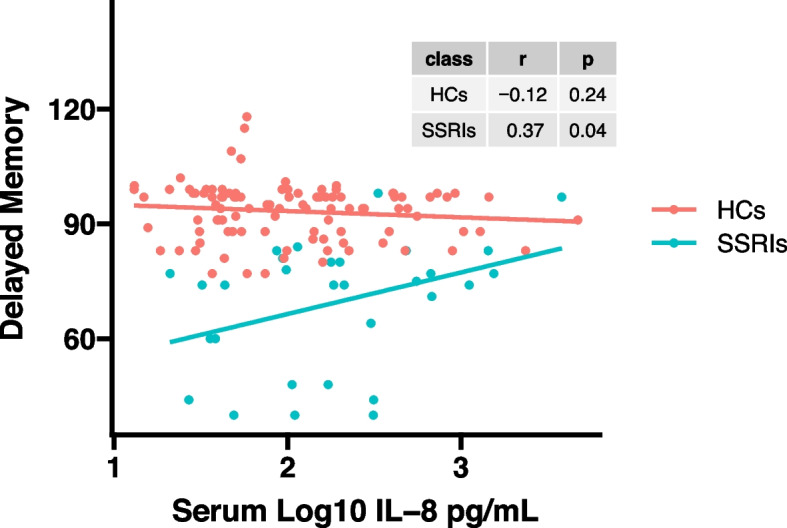
A positive correlation between serum log_10_IL-8 levels and delayed memory score was found in MDD patients with SSRIs (*r* = 0.37, *p* = 0.04), but not in healthy controls (*r* = -0.12, *p* = 0.24)

**Fig. 3:**
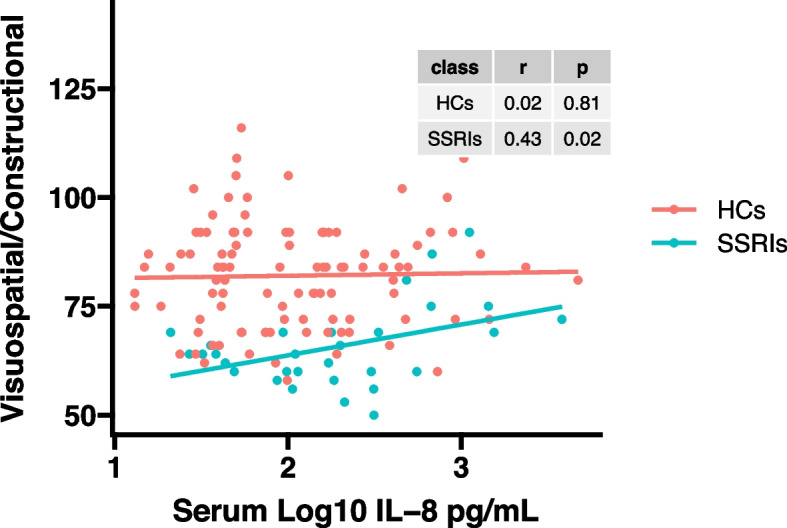
A positive correlation between serum log_10_IL-8 levels and visuospatial/constructional score was found in MDD patients with SSRIs (*r* = 0.43, *p* = 0.02), but not in healthy controls (*r* = 0.02, *p* = 0.81)

As shown in Figs. 2 and 3, serum log_10_IL-8 levels were significantly correlated with delayed memory score (*r* = 0.37, *p* = 0.04) and visuospatial/constructional score (*r* = 0.43, *p* = 0.02) in MDD patients by Pearson correlation analysis. However, significant correlations were not observed in healthy controls (delayed memory score: *r* = -0.12, *p* = 0.24; visuospatial/constructional score: *r* = 0.02, *p* = 0.81). In addition, there were no significant correlations between serum log_10_IL-8 levels and other RBANS test scores in MDD patients with SSRIs as well as in healthy controls. The correlation analysis results are presented in Supplemental Table S1.

## Discussion

To our knowledge, this is the first cross-sectional study that investigated serum IL-8 levels, cognitive function, and their associations in MDD patients with SSRIs. This study had three major findings: 1) cognitive function was worse in MDD patients with SSRIs than in healthy controls; 2) serum log_10_IL-8 levels were nominally higher in MDD patients with SSRIs than in healthy controls; 3) serum log_10_IL-8 levels were positively associated with delayed memory and visuospatial/constructional sub-scores in MDD patients with SSRIs.

Increasing evidence from previous studies has supported that cognitive impairment is a core feature of MDD and it affects the treatment, rehabilitation, quality life, and even employment for MDD [1–3, 7]. Thus, mitigating cognitive deficits should be the focus of MDD treatment [8, 10]. Our data showed that all RBANS test scores were significantly lower in patients with MDD than in healthy controls, suggesting that MDD patients still exhibited poorer cognitive abilities even though they were treated with the SSRI antidepressants. This finding was supported by a recent study that reported significant differences in all RBANS test scores between 116 healthy controls and 90 MDD patients treated with anti-depressants (including 39 patients with SSRIs) [6]. Several previous studies also demonstrated that there was a marked decline in the RBANS total score in MDD patients in comparison to healthy controls [3, 36–39]. In addition, several recent studies reported that the SSRIs could improve cognitive function in patients with MDD [10, 40]. Although patients with MDD were administrated oral single SSRI in the present cross-sectional study, our data did not support that SSRIs could achieve cognitive remission of MDD. Thus, a longitudinal and multicenter follow-up SSRI intervention study should be performed to validate the effect of SSRI treatment on cognitive performance of MDD in the future.

IL-8 was an inflammatory cytokine that was produced by macrophages and brain neuron cells such as microglia and astrocytes [11, 12]. IL-8 may be implicated in the pathogenesis of psychiatric disorders and their treatment effect [41]. Our finding showed that serum log_10_IL-8 levels were nominally elevated in MDD patients with SSRIs than in healthy controls, which further suggest that higher IL-8 levels might be involved in the psychopathology of depression. Previous studies demonstrated that increased serum IL-8 levels were implicated in the underlying pathogenesis of MDD. For example, at the protein level, cerebrospinal fluid (CSF)/serum/plasma levels of IL-8 were significantly increased in patients with MDD in comparison to healthy controls [42–46]. At the molecular level, a higher mRNA expression level of the *IL-8* gene (*IL-8*, located on chromosome 4q) was reported in the prefrontal cortex of drug-free MDD patients compared to healthy controls [47]. Moreover, the rs4078 polymorphism in *IL-8* was significantly associated with the susceptibility to MDD [48, 49]. However, the mRNA and protein levels of *IL-8* were found significantly declined in patients with MDD in comparison to healthy controls [50, 51]. In addition, several studies have reported that there were no significant differences in the protein and mRNA levels of *IL-8* between patients with MDD and healthy controls [52–55]. These discrepant results may be due to the effects of a series of confounding factors, such as sex, age, BMI, age of onset, illness duration, antidepressant types and dosages, diet, sample size, and ethnicities.

SSRI antidepressants have been reported to activate microglia and astrocytes to synthesize and release inflammatory cytokines [15–20]. Dysfunctional microglia and astrocytes in the hippocampus were implicated in cognitive deficits in individuals with brain disorders [21]. The above evidence hinted that there might be significant associations among inflammation cytokines, cognitive function, SSRIs, and microglia and astrocytes. Intriguingly, our study found that serum log_10_IL-8 levels were nominally elevated in MDD patients with SSRIs than in healthy controls, further suggesting that elevated IL-8 levels in patients with MDD might be modulated by SSRIs which activated the microglia and astrocytes of depression. Moreover, serum log_10_IL-8 levels were positively associated with the sub-scores of RBANS delayed memory and visuospatial/constructional function in MDD patients with SSRIs, hinting that a higher concentration of serum IL-8 corresponds to improving MDD delayed memory and visuospatial/constructional by SSRIs modulating the activity of microglia and astrocytes to synthesize and release IL-8. This finding also indicated that elevated serum IL-8 levels might have neuroprotection to the brains of MDD [56, 57]. Previous studies demonstrated that a decline in serum/plasma IL8 levels was implicated in cognitive impairment disorders including MCI and AD [27, 29]. However, the neurotoxic effect of IL-8 on human brain function has also been reported. Increased serum IL-8 levels were involved in poor performance in memory, cognitive speed and motor function in normal elderly subjects [26]. Thus, the effect of IL-8 on cognitive function of brain disorders might be complex, i.e., IL-8 has either neuroprotective or neurotoxic roles [56, 58]. However, our finding has shown that elevated IL-8 levels might improve cognitive function in MDD patients following the administration of oral single SSRI. Thus, further longitudinal studies should be designed to explore the relationships among serum IL-8 levels, cognition, and SSRIs in first-episode drug-free patients with MDD.

Several limitations of the present study should be interpreted as follows: 1) *A relatively small sample size*. Our results should be considered a pilot study; 2) *Comparison of IL-8 levels between patients with SSRIs and Healthy controls.* Thus, it is unclear whether serum IL-8 levels are higher due to depression or the use of SSRIs; 3) *A cross-sectional design*. It was unclear whether there was a causative association between increased serum log_10_IL-8 levels and cognitive improvement in MDD patients with SSRIs. Thus, future longitudinal and prospective follow-up studies are necessary to clarify the relationships among serum log_10_IL-8 levels, cognition and SSRIs in first-episode drug-free patients with MDD; 4) *Confirmed MDD diagnosis*. Although patients were diagnosed as having unipolar depression rather than bipolar depression during enrollment, a few unipolar depressive patients may develop bipolar depression later; and 5) *Types and dosages of SSRIs*. Future studies should also examine the effect of different SSRI types and dosages on serum IL-8 levels and cognitive function in MDD patients.

## Conclusions

Serum log_10_IL-8 levels were nominally higher and cognitive impairment was more severe in MDD patients with SSRIs in comparison to healthy controls. Serum log_10_IL-8 levels were positively associated with the sub-scores of RBANS delayed memory and visuospatial/constructional function in MDD patients. Our data from the present cross-sectional study further revealed that increased serum IL-8 levels might be implicated in the MDD psychopathology or the use of SSRIs, and elevated serum IL-8 levels contributed to improving delayed memory and visuospatial/constructional function of MDD, and cognitive function in patients with SSRIs was impaired in comparison to healthy controls. However, the present findings were preliminary due to the relatively small sample size and the absence of a longitude follow-up. Therefore, future studies are warranted to confirm the present findings using a large, independent and SSRI invention cohort of first-episode drug-free patients with MDD.

## Supplementary Information

**Additional file 1:** **Supplementary Table 1.** The correlations between serum log_10_IL-8 levels and all RBANS test scores in MDD patients with SSRIs and healthy controls.

## Data Availability

All authors have confirmed that all data and materials as well as software application support their published claims and comply with the field standards. Moreover, the datasets used and/or analyzed during the current study available from the corresponding author on reasonable request.

## References

[1] Lee RSC, Hermens DF, Porter MA, Redoblado-Hodge MA (2012). A meta-analysis of cognitive deficits in first-episode Major Depressive Disorder. J Affect Disord.

[2] Ye G, Yin GZ, Tang Z (2018). Association between increased serum interleukin-6 levels and sustained attention deficits in patients with major depressive disorder. Psychol Med.

[3] Hu WM, Yin XY, Yin XL (2020). Prevalence, social-demographic and cognitive correlates of depression in Chinese psychiatric medical staff. J Affect Disord.

[4] Snyder HR (2013). Major depressive disorder is associated with broad impairments on neuropsychological measures of executive function: A meta-analysis and review. Psychol Bull.

[5] Corsi MM, Licastro F, Porcellini E (2011). Reduced plasma levels of P-selectin and L-selectin in a pilot study from Alzheimer disease: relationship with neuro-degeneration. Biogerontology.

[6] Hou WL, Yin XL, Yin XY, et al. Association between stereopsis deficits and attention decline in patients with major depressive disorder. Prog Neuropsychopharmacol Biol Psychiatry. 2021;110:110267 10/gk7qtq. 10.1016/j.pnpbp.2021.110267 33556482

[7] Millan MJ, Agid Y, Brüne M (2012). Cognitive dysfunction in psychiatric disorders: characteristics, causes and the quest for improved therapy. Nat Rev Drug Discov.

[8] Hagen BI, Lau B, Joormann J, Småstuen MC, Landrø NI, Stubberud J (2020). Goal management training as a cognitive remediation intervention in depression: A randomized controlled trial. J Affect Disord.

[9] Listunova L, Kienzle J, Bartolovic M (2020). Cognitive remediation therapy for partially remitted unipolar depression: A single-blind randomized controlled trial. J Affect Disord.

[10] Sagud M, Nikolac Perkovic M, Dvojkovic A (2021). Distinct association of plasma BDNF concentration and cognitive function in depressed patients treated with vortioxetine or escitalopram. Psychopharmacology.

[11] Ehrlich LC, Hu S, Sheng WS (1998). Cytokine regulation of human microglial cell IL-8 production. J Immunol.

[12] Remick DG (2005). Interleukin-8. Crit Care Med.

[13] Kronfol Z (2000). Cytokines and the Brain: Implications for Clinical Psychiatry. Am J Psychiatry.

[14] Licinio J, Wong ML, Gold PW (1992). Neutrophil-activating peptide-1 /interleukin-8 mRNA is localized in rat hypothalamus and hippocampus. NeuroReport.

[15] Hashioka S, Klegeris A, Monji A (2007). Antidepressants inhibit interferon-γ-induced microglial production of IL-6 and nitric oxide. Exp Neurol.

[16] Hashioka S, McGeer P, Monji A, Kanba S (2009). Anti-Inflammatory Effects of Antidepressants: Possibilities for Preventives Against Alzheimers Disease. CNSAMC.

[17] Hwang J, Zheng LT, Ock J (2008). Inhibition of glial inflammatory activation and neurotoxicity by tricyclic antidepressants. Neuropharmacology.

[18] Horikawa H, Kato TA, Mizoguchi Y (2010). Inhibitory effects of SSRIs on IFN-γ induced microglial activation through the regulation of intracellular calcium. Prog Neuropsychopharmacol Biol Psychiatry.

[19] Chen B, Zhang M, Ji M (2021). The Association Between Antidepressant Effect of SSRIs and Astrocytes: Conceptual Overview and Meta-analysis of the Literature. Neurochem Res.

[20] Borroto-Escuela DO, Ambrogini P, Narvaez M (2021). Serotonin Heteroreceptor Complexes and Their Integration of Signals in Neurons and Astroglia—Relevance for Mental Diseases. Cells.

[21] Ryan SM, Nolan YM (2016). Neuroinflammation negatively affects adult hippocampal neurogenesis and cognition: can exercise compensate?. Neurosci Biobehav Rev.

[22] Khansari PS, Sperlagh B (2012). Inflammation in neurological and psychiatric diseases. Inflammopharmacol.

[23] Réus GZ, Fries GR, Stertz L (2015). The role of inflammation and microglial activation in the pathophysiology of psychiatric disorders. Neuroscience.

[24] Gałecki P, Mossakowska-Wójcik J, Talarowska M (2018). The anti-inflammatory mechanism of antidepressants – SSRIs, SNRIs. Prog Neuropsychopharmacol Biol Psychiatry.

[25] Wang L, Wang R, Liu L, Qiao D, Baldwin DS, Hou R (2019). Effects of SSRIs on peripheral inflammatory markers in patients with major depressive disorder: A systematic review and meta-analysis. Brain Behav Immun.

[26] Baune BT, Ponath G, Golledge J (2008). Association between IL-8 cytokine and cognitive performance in an elderly general population—The MEMO-Study. Neurobiol Aging.

[27] Kim SM, Song J, Kim S (2011). Identification of peripheral inflammatory markers between normal control and Alzheimer’s disease. BMC Neurol.

[28] Alsadany MA, Shehata HH, Mohamad MI, Mahfouz RG (2013). Histone Deacetylases Enzyme, Copper, and IL-8 Levels in Patients With Alzheimer’s Disease. Am J Alzheimers Dis Other Demen.

[29] Hesse R, Wahler A, Gummert P (2016). Decreased IL-8 levels in CSF and serum of AD patients and negative correlation of MMSE and IL-1β. BMC Neurol.

[30] Zhu Y, Chai YL, Hilal S (2017). Serum IL-8 is a marker of white-matter hyperintensities in patients with Alzheimer’s disease. Alzheimers Dement (Amst).

[31] Zhu ZH, Song XY, Man LJ (2022). Comparisons of serum interleukin-8 levels in major depressive patients with drug-free versus SSRIs versus healthy control. Front Psychiatry.

[32] Randolph C, Tierney MC, Mohr E, Chase TN (1998). The Repeatable Battery for the Assessment of Neuropsychological Status (RBANS): preliminary clinical validity. J Clin Exp Neuropsychol.

[33] Guan LY, Hou WL, Zhu ZH (2021). Associations among gonadal hormone, triglycerides and cognitive decline in female patients with major depressive disorders. J Psychiatr Res.

[34] Hou W, Yin X, Yin X (2021). Association between stereopsis deficits and attention decline in patients with major depressive disorder. Prog Neuropsychopharmacol Biol Psychiatry.

[35] Zhang BH, Tan YL, Zhang WF (2008). Repeatable battery for the assessment of neuropsychological status as a screening test in Chinese: reliability and validity. Chin Ment Health J.

[36] Baune BT, Miller R, McAfoose J, Johnson M, Quirk F, Mitchell D (2010). The role of cognitive impairment in general functioning in major depression. Psychiatry Res.

[37] Shao TN, Yin GZ, Yin XL (2017). Elevated triglyceride levels are associated with cognitive impairments among patients with major depressive disorder. Compr Psychiatry.

[38] Hui L, Han M, Du XD (2017). Serum ApoB levels in depressive patients: associated with cognitive deficits. Sci Rep.

[39] Jia QF, Chen P, Zhu HL (2019). Cognitive Impairments in First-Episode Drug-Naïve Versus Medicated Depressive Patients: RBANS in a Chinese Population. Psychiatr Q.

[40] Liu L, Lv X, Zhou S (2021). The effect of selective serotonin reuptake inhibitors on cognitive impairment in patients with depression: A prospective, multicenter, observational study. J Psychiatr Res.

[41] Tsai SJ (2021). Role of interleukin 8 in depression and other psychiatric disorders. Prog Neuropsychopharmacol Biol Psychiatry.

[42] Baune BT, Smith E, Reppermund S (2012). Inflammatory biomarkers predict depressive, but not anxiety symptoms during aging: The prospective Sydney Memory and Aging Study. Psychoneuroendocrinology.

[43] Dahl J, Ormstad H, Aass HCD (2014). The plasma levels of various cytokines are increased during ongoing depression and are reduced to normal levels after recovery. Psychoneuroendocrinology.

[44] Vogelzangs N, de Jonge P, Smit JH, Bahn S, Penninx BW (2016). Cytokine production capacity in depression and anxiety. Transl Psychiatry.

[45] Kim JM, Stewart R, Kim JW (2018). Changes in pro-inflammatory cytokine levels and late-life depression: A two year population based longitudinal study. Psychoneuroendocrinology.

[46] Wang AK, Miller BJ (2018). Meta-analysis of Cerebrospinal Fluid Cytokine and Tryptophan Catabolite Alterations in Psychiatric Patients: Comparisons Between Schizophrenia, Bipolar Disorder, and Depression. Schizophr Bull.

[47] Shelton RC, Claiborne J, Sidoryk-Wegrzynowicz M (2011). Altered expression of genes involved in inflammation and apoptosis in frontal cortex in major depression. Mol Psychiatry.

[48] Kim JM, Stewart R, Kim SW (2013). Physical health and incident late-life depression: modification by cytokine genes. Neurobiol Aging.

[49] Reyes-Gibby CC, Wang J, Spitz M, Wu X, Yennurajalingam S, Shete S (2013). Genetic Variations in Interleukin-8 and Interleukin-10 Are Associated With Pain, Depressed Mood, and Fatigue in Lung Cancer Patients. J Pain Symptom Manage.

[50] Pantazatos SP, Huang YY, Rosoklija GB, Dwork AJ, Arango V, Mann JJ (2017). Whole-transcriptome brain expression and exon-usage profiling in major depression and suicide: evidence for altered glial, endothelial and ATPase activity. Mol Psychiatry.

[51] Çakici N, Sutterland AL, Penninx BWJH, Dalm VA, de Haan L, van Beveren NJM (2020). Altered peripheral blood compounds in drug-naïve first-episode patients with either schizophrenia or major depressive disorder: a meta-analysis. Brain Behav Immun.

[52] Dowlati Y, Herrmann N, Swardfager W (2010). A Meta-Analysis of Cytokines in Major Depression. Biol Psychiat.

[53] Eyre HA, Air T, Pradhan A (2016). A meta-analysis of chemokines in major depression. Prog Neuropsychopharmacol Biol Psychiatry.

[54] Köhler CA, Freitas TH, Maes M (2017). Peripheral cytokine and chemokine alterations in depression: a meta-analysis of 82 studies. Acta Psychiatr Scand.

[55] Pandey GN, Rizavi HS, Zhang H, Ren X (2018). Abnormal gene and protein expression of inflammatory cytokines in the postmortem brain of schizophrenia patients. Schizophr Res.

[56] Semple BD, Kossmann T, Morganti-Kossmann MC (2010). Role of Chemokines in CNS Health and Pathology: A Focus on the CCL2/CCR2 and CXCL8/CXCR2 Networks. J Cereb Blood Flow Metab.

[57] Ryu JK, Cho T, Choi HB, Jantaratnotai N, McLarnon JG (2015). Pharmacological antagonism of interleukin-8 receptor CXCR2 inhibits inflammatory reactivity and is neuroprotective in an animal model of Alzheimer’s disease. J Neuroinflammation.

[58] Franciosi S, Choi HB, Kim SU, McLarnon JG (2005). IL-8 enhancement of amyloid-beta (Aβ1-42)-induced expression and production of pro-inflammatory cytokines and COX-2 in cultured human microglia. J Neuroimmunol.

