# Supplementary material for: Association between increased serum interleukin-8 levels and improved cognition in major depressive patients with SSRIs
Source: BMC Psychiatry. 2023 Feb 23;23:122. doi: 10.1186/s12888-023-04616-z (PMC9948487; doi:10.1186/s12888-023-04616-z)
Supplement: Supplementary file 1 — Additional file 1: Supplementary Table 1. The correlations between serum log10IL-8 levels and all RBANS test scores in MDD patients with SSRIs and healthy controls. [file 12888_2023_4616_MOESM1_ESM.docx]

**Supplementary Table 1.** The correlations between serum log_10_IL-8 levels and all RBANS test scores in MDD patients with SSRIs and healthy controls

|  | MDD Patients with SSRIs | | Healthy Controls | |
| --- | --- | --- | --- | --- |
| Index | r | *p* | r | *p* |
| Immediate Memory | 0.12 | 0.35 | -0.02 | 0.81 |
| Visuospatial/Constructional | 0.43 | **0.02** | 0.02 | 0.81 |
| Language | 0.10 | 0.61 | -0.18 | 0.07 |
| Attention | -0.35 | 0.06 | 0.01 | 0.91 |
| Delayed Memory | 0.37 | **0.04** | -0.12 | 0.24 |
| RBANS Total Score | 0.15 | 0.44 | -0.09 | 0.35 |
